# Singular Case of Carditis

**Published:** 1811-05

**Authors:** 


*For the Medical and Physical Journal.*

### *Singular Case of Carditis.*

We are indebted to Andrew Baird, M. D. Inspector of Naval Hospitals, &c. &c. &c. for this extraordinary Case. It is to be regretted that the history of the symptoms and progress of the disease extends only to four days previous to the death of the patient.

Editor.

(CASE.)

Mr. ———, late Gunner of the ship Neptune, was admitted into the Royal Hospital of Plymouth, as a physician's patient, on the 23d of January, in the present year. He complained of loss of strength, cough accompanied with some expectoration, slight pain of the breast, difficulty of respiration in the recumbent position; tongue white, thirst urgent, skin hot and dry; pulse small, quick and hard; bowels open. The gentleman under whose care he had been previous to admission into the hospital, furnished no other history of the case than the following. He found the patient with symptoms of fever, violent head-ach, nausea, and frequent rigors: the only medicine given to him was an emetic.

When

When received into the hospital he was bled largely, and the bowels freely evacuated, by which the pain in the breast and difficulty of respiration were in some degree moderated; in other respects he continued nearly in the same state until the morning of the 27th, when he suddenly expired.

(*Inspectio Cadaveris.*)

On raising the sternum the pericardium was found adhering in many places, and considerably distended. In the cavity of the thorax there was a considerable quantity of dark watery blood, in which no coagulum could be discovered; and on puncturing the pericardium, about half an ounce of fluid of the same appearance issued out. An attempt was made to discover an opening in the pericardium communicating with the chest, through which this fluid might have passed, but without effect.

The dissection had proceeded thus far, when the relations of the deceased demanding the body for interment, the heart and pericardium were suddenly removed, and delivered to Mr. Hammick, senior Surgeon of the hospital, who proceeding with the inspection, states, that the pericardium (which was very thin, but in many parts of its surface loaded with fat,) bore evident marks of inflammation, it being crowded with small vessels, more particularly on its anterior part, where it had almost the appearance of mortification. The heart itself was much enlarged, was entirely enveloped in layers of dark coagulable lymph, thrown out in the form of a net-work, and was connected, by slight adhesions, to different parts of the inner surface of the pericardium. On removing the coagulable lymph, the surface of the heart, together with the large vessels, appeared excessively inflamed. About two inches and an half from the apex of the heart there was a small ulcerated spot of a brownish colour, the size of a sixpence, but superficial, and half an inch below this spot was observed a pin, which was found, upon further examination, embedded in the muscular parieties of the heart itself. This pin was of a common size, but without a head. It lay in an ulcerated groove, with the point inwards, and the end of the shaft, which was a little bent, projecting about a line beyond the external surface. It had entered the heart on the anterior surface of the obtuse margin, about two inches above the apex, opposite to the *septum cordis*, from whence it passed in an oblique direction upwards and to the right side, its point lying just within the cavity of the right ventricle.

